# Leukotrienes Are Upregulated and Associated with Human T-Lymphotropic Virus Type 1 (HTLV-1)-Associated Neuroinflammatory Disease

**DOI:** 10.1371/journal.pone.0051873

**Published:** 2012-12-20

**Authors:** Bruno Caetano Trindade, Carlos Artério Sorgi, Larissa Deadame de Figueiredo Nicolete, Tathiane Maistro Malta, Mariana Tomazini Pinto, Osvaldo Massaiti Takayanagui, Dimas Tadeu Covas, Olindo Assis Martins Filho, Simone Kashima, Lúcia Helena Faccioli

**Affiliations:** 1 Laboratório de Inflamação e Imunologia das Parasitoses, Faculdade de Ciências Farmacêuticas de Ribeirão Preto, Universidade de São Paulo, Ribeirão Preto, Sao Paulo, Brazil; 2 Laboratório de Biomarcadores de Diagnóstico e Monitoração, Centro de Pesquisas René Rachou, Fundação Oswaldo Cruz, Belo Horizonte, Brazil; 3 Instituto Nacional de Ciência e Tecnologia em Células-Tronco e Terapia Celular/Hemocentro de Ribeirão Preto, Ribeirão Preto, Sao Paulo, Brazil; 4 Departamento de Neurociências e Ciências do Comportamento, Faculdade de Medicina de Ribeirão Preto, Universidade de São Paulo, Ribeirão Preto, Sao Paulo, Brazil; 5 Departamento de Clínica Médica, Faculdade de Medicina de Ribeirão Preto, Universidade de São Paulo, Ribeirão Preto, Sao Paulo, Brazil; Blood Systems Research Institute, United States of America

## Abstract

Leukotrienes (LTs) are lipid mediators involved in several inflammatory disorders. We investigated the LT pathway in human T-lymphotropic virus type 1 (HTLV-1) infection by evaluating LT levels in HTLV-1-infected patients classified according to the clinical status as asymptomatic carriers (HACs) and HTLV-1-associated myelopathy/tropical spastic paraparesis (HAM/TSP) patients. Bioactive LTB_4_ and CysLTs were both increased in the plasma and in the supernatant of peripheral blood mononuclear cell cultures of HTLV-1-infected when compared to non-infected. Interestingly, CysLT concentrations were increased in HAM/TSP patients. Also, the concentration of plasma LTB_4_ and LTC_4_ positively correlated with the HTLV-1 proviral load in HTLV-1-infected individuals. The gene expression levels of LT receptors were differentially modulated in CD4^+^ and CD8^+^ T cells of HTLV-1-infected patients. Analysis of the overall plasma signature of immune mediators demonstrated that LT and chemokine amounts were elevated during HTLV-1 infection. Importantly, in addition to CysLTs, IP-10 was also identified as a biomarker for HAM/TSP activity. These data suggest that LTs are likely to be associated with HTLV-1 infection and HAM/TSP development, suggesting their putative use for clinical monitoring.

## Introduction

Human T-lymphotropic virus type 1 (HTLV-1), a complex retrovirus, is the causal agent of adult T cell leukemia (ATL), HTLV-1-associated myelopathy/tropical spastic paraparesis (HAM/TSP) and other inflammatory disorders that develop after a variable period of latency ranging between months and decades [1], [2]. Although the majority of HTLV-1-infected individuals remain asymptomatic carriers (HACs), the lifetime risk of developing HTLV-1-associated diseases may be close to 10%, and the incidence of HAM/TSP ranges from 0.3% to 4% [3].

HAM/TSP is a neuroinflammatory disease characterized by a chronic progressive myelopathy with infiltrating mononuclear cells in the areas of demyelination and axonal dystrophy [4], [5]. It is not clear how HTLV-1 causes neurological damage, but spontaneous T cell proliferation and proinflammatory responses characterized by elevated ex vivo production of interferon (IFN)-γ and tumor necrosis factor (TNF)-α by peripheral blood mononuclear cells (PBMCs) are associated with HAM/TSP [6], [7]. In addition, patients with HAM/TSP display an increased proviral burden when compared to HACs, and high proviral loads have been associated with rapid disease progression [8]–[10]. Thus, few disease markers and prognostic predictors have been described for HAM/TSP.

Leukotrienes (LTs) are bioactive lipid mediators involved with inflammatory conditions [11] that may represent candidate biomarkers for HAM/TSP. Biosynthesis of LTs is triggered by stimuli such as antigen, cytokines, microorganisms and immune complexes [12]. Just after stimulation, arachidonic acid (AA) that is liberated from cellular membrane phospholipids through the action of phospholipase A_2_ (PLA_2_) is oxidized by 5-lipoxygenase (5-LO) in combination with 5-LO-activating protein (FLAP) to generate the leukotriene A_4_ (LTA_4_). The downstream enzymes LTA_4_ hydrolase (LTA_4_H) and LTC_4_ synthase (LTC_4_S) give rise to leukotriene B_4_ (LTB_4_) and leukotriene C_4_ (LTC_4_). LTC_4_ is further converted to LTD_4_ and LTE_4_, which are collectively termed cysteinyl leukotrienes (CysLTs) respectively. LTB_4_ and CysLTs signal through distinct cell surface receptors named BLT_1_ and BLT_2_ and CysLT_1_ and CysLT_2_, respectively [13]. Functionally, LTB_4_ is recognized as a potent leukocyte chemoattractant that also displays leukocyte activating functions, whereas the CysLT are better known for leading to airway constriction, increased vascular permeability, mucus secretion and cell trafficking [14]. In addition, LTs have been shown to improve the host defense against pathogens [15]–[18].

Considering the importance of LTs as powerful mediators of inflammation, the present study was undertaken to test the hypothesis that HTLV-1 infection leads to an exacerbation of the 5-LO products formation and LT signaling in patients with HAM/TSP. We examined LT concentrations in plasma, the ability of PBMCs to produce LTs and LT receptor expression in lymphocytes from HTLV-1 patients. We also investigated the overall plasma LT, chemokine and cytokine signatures of HACs and HAM/TSP patients. Moreover, we investigated the correlations between LTs, chemokines and cytokines in HTLV-1-infected individuals and the capacity of LTs to modulate cytokine production. Our results demonstrate for the first time that LTs are upregulated during HTLV-1 infection, suggesting a role for LTs in HAM/TSP pathogenesis and presenting them as potential biomarkers for monitoring HAM/TSP development.

## Results

### CysLT is Upregulated in HTLV-1-associated Neuroinflammatory Disease

**Figure 1:**
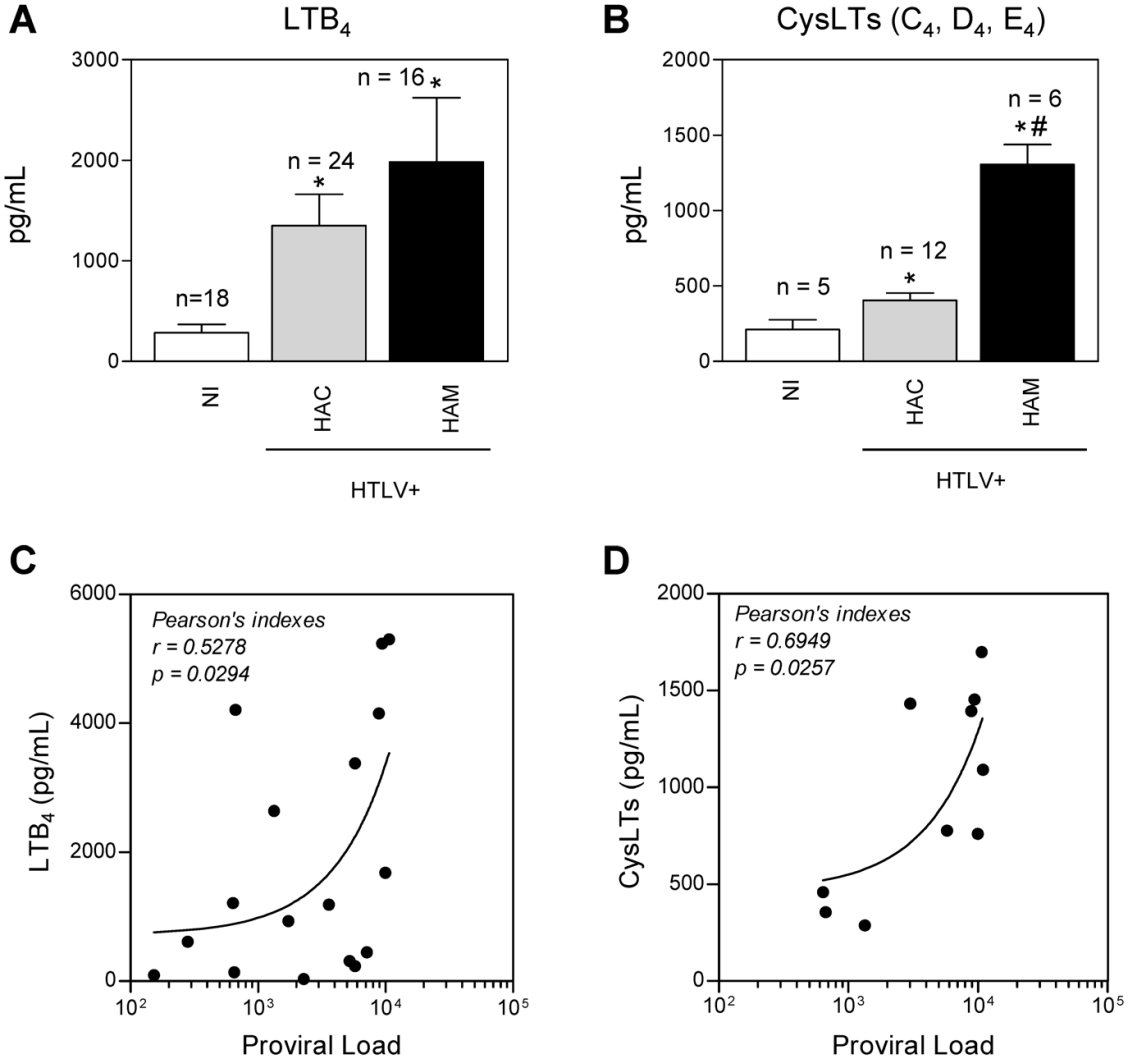
Leukotrienes are increased in the plasma of HTLV-1-infected subjects and correlate with the proviral load. LTB_4_ (A) and CysLTs (B) were measured by EIA after sample purifications on Waters C18 Sep-Pak cartridges. Plasma from non-infected healthy donors (NI), HTLV-1 asymptomatic carriers (HAC) and HAM/TSP subjects (HAM) were used. (C, D) A correlation is shown between the plasma levels of LTB_4_ (C), CysLTs (D) and the DNA proviral load in peripheral blood mononuclear cells of HTLV-1-infected individuals (HAC and HAM/TSP) using Pearson rank correlation test. The data are presented as means ± SEM. *p<0.05, compared with control; ^#^ *p*<0.05, compared with HAC (one-way ANOVA). Statistically significant correlations at *p*<0.05 are displayed in the graphs along with Pearson’s coefficient (r).

LTs have been shown to function as inflammatory mediators [11]. To investigate whether HAM/TSP disease is characterized by elevated levels of LTs, we measured the amount of these mediators in the plasma of HTLV-1 patients. LTB_4_ was increased in the plasma of HACs and HAM/TSP patients when compared to that of NI donors; however, no difference was observed in LTB_4_ levels between HACs and HAM/TSP patients (Figure 1A). Interestingly, HACs and HAM/TSP patients displayed increased amounts of CysLTs when compared with NI donors, but CysLT amounts were higher in the plasma of HAM/TSP patients than in the plasma of HACs (Figure 1B). Thus, although HTLV-1 induces increased concentrations of LTs in the plasma of both HACs and HAM/TSP patients, these results associate increased CysLT concentrations with HAM/TSP. In addition, we explored the correlation between HTLV-1 proviral load and plasma LTB_4_ (Figure 1C) or CysLTs (Figure 1D) and found a positive correlation. Thus, in infected persons, the plasma LTs are associated with the HTLV-1 proviral load in PBMCs.

### HTLV-1 Enhances LT Generation

**Figure 2:**
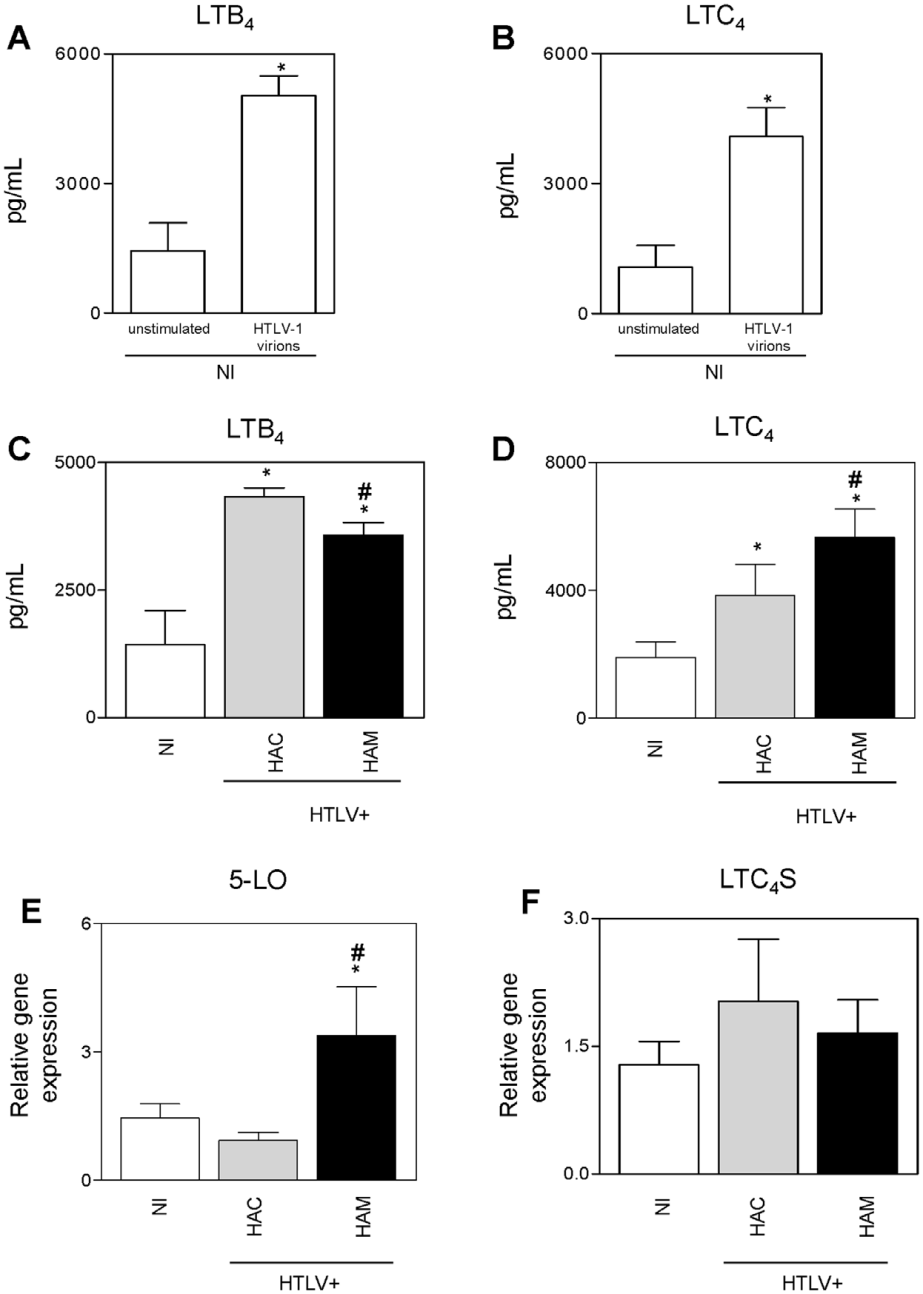
HTLV-1 primes cells for leukotriene generation. (A–D) 10^6^ peripheral blood mononuclear cells were cultured for 48 hours and then treated for 30 min with A23187 (0.5 µM) before detection of LTB_4_ (A, C) and LTC_4_ (B, D) by EIA (n = 7 per group). (A, B) Cells from non-infected healthy donors were seeded alone (unstimulated) or in the presence of cell-free HTLV-1 (HTLV-1 virions). (C, D) Cells from non-infected healthy donors (NI), HTLV-1 asymptomatic carriers (HAC) and HAM/TSP patients (HAM) were cultured. (E,F) Quantitative PCR (qPCR) was performed to detect 5-LO (E) and LTC_4_ synthase (F). The relative expression levels of these genes were determined in PBMCs from NI donors, HACs and HAM/TSP patients (n = 15 per group). Gene expression levels were normalized to the expression level of GAPDH mRNA in the same real-time PCR reaction. The data are presented as means ± SEM. *p<0.05, compared with unstimulated samples or NI donors; ^#^ *p*<0.05, compared with HACs (*t*-test or one-way ANOVA as appropriate).

HTLV-1-induced LT generation was examined in PBMCs of NI donors. We found increased production of LTB_4_ (Figure 2A) and LTC_4_ (Figure 2B) when cells were challenged with cell-free virus. Next, because we found increased levels of LTs in the plasma of HTLV-1 patients, we measured LT generation by PBMCs from HTLV-1 patients. We observed increased production of LTB_4_ by cells from HACs and HAM/TSP patients when compared to those from NI donors, with the highest amount of LTB_4_ in the supernatant of cells from HACs (Figure 2C). Moreover, as expected, the generation of CysLTs was increased in PBMCs from HACs and HAM/TSP patients when compared to those from NI donors, with the highest amount of CysLTs in cells from HAM/TSP patients (Figure 2D). Next, we assessed 5-LO and LTC_4_ synthase expression in PBMCs. Our results demonstrated that cells from HAM/TSP patients expressed higher levels of 5-LO than cells from NI donors or HACs (Figure 2E); however, no differences in the expression of LTC_4_ synthase were observed between the groups (Figure 2F).

### Lymphocytes from HTLV-1 Patients have Altered LT Receptor Gene Expression

**Figure 3:**
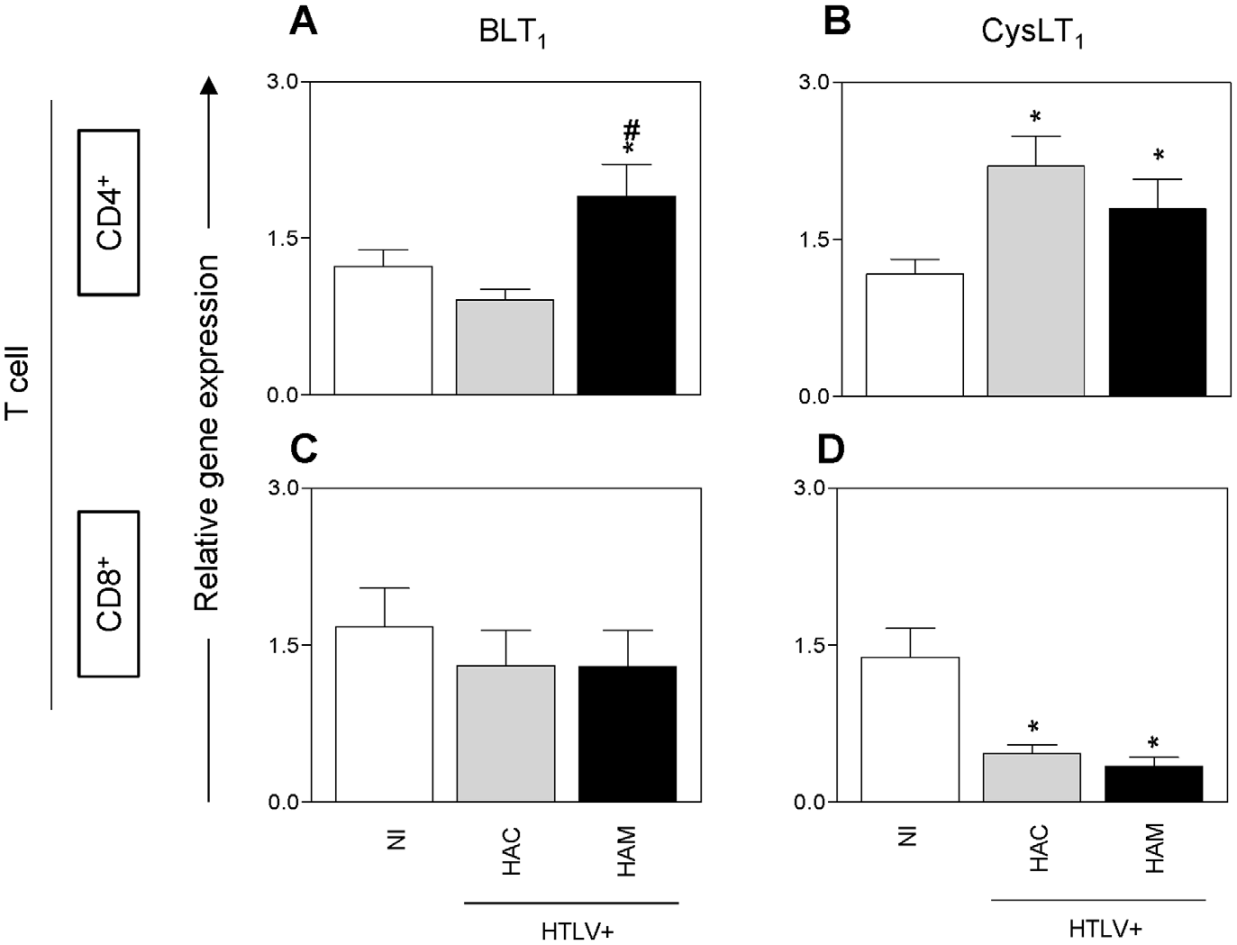
Leukotriene receptor mRNA expression in lymphocytes of HTLV-1 patients. Quantitative PCR (qPCR) was performed for BLT_1_ (A, C) and CysLT_1_ (B, D), and their relative expression levels were determined in CD4^+^ (A, B) and CD8^+^ (C, D) T cells from twenty non-infected healthy donors (NI), twenty asymptomatic carriers (HAC) and seventeen HAM/TSP patients (HAM). Gene expression levels were normalized to the gene expression levels of *ACTB, GAPDH, B2M* and *RPL13a* for CD4^+^ T cells and of *ACTB* for CD8^+^ T cells in the same real-time PCR reaction. The data are presented as means ± SEM. *p<0.05, compared with NI donors; ^#^ *p*<0.05, compared with HACs (one-way ANOVA).

We next analyzed the gene expression of LT receptors by detecting BLT_1_ and CysLT_1_ expression in both CD4^+^ and CD8^+^ T cells (Figure 3). In CD4^+^ T cells, BLT_1_ expression was increased only in HAM/TSP patients than in NI donors (Figure 3A) but CysLT_1_ was expressed at higher amounts in HACs and HAM/TSP patients than in NI donors (Figure 3B). Analysis of CD8^+^ T cells showed no differences in BLT_1_ gene expression among all of the groups (Figure 3C), whereas decreased CysLT_1_ gene expression was detected in HAC and HAM/TSP patient CD8^+^ T cells when compared to NI donor CD8^+^ T cells (Figure 3D).

### Overall Plasma Signatures of LTs, Chemokines and Cytokines in HTLV-1 Infection

**Figure 4:**
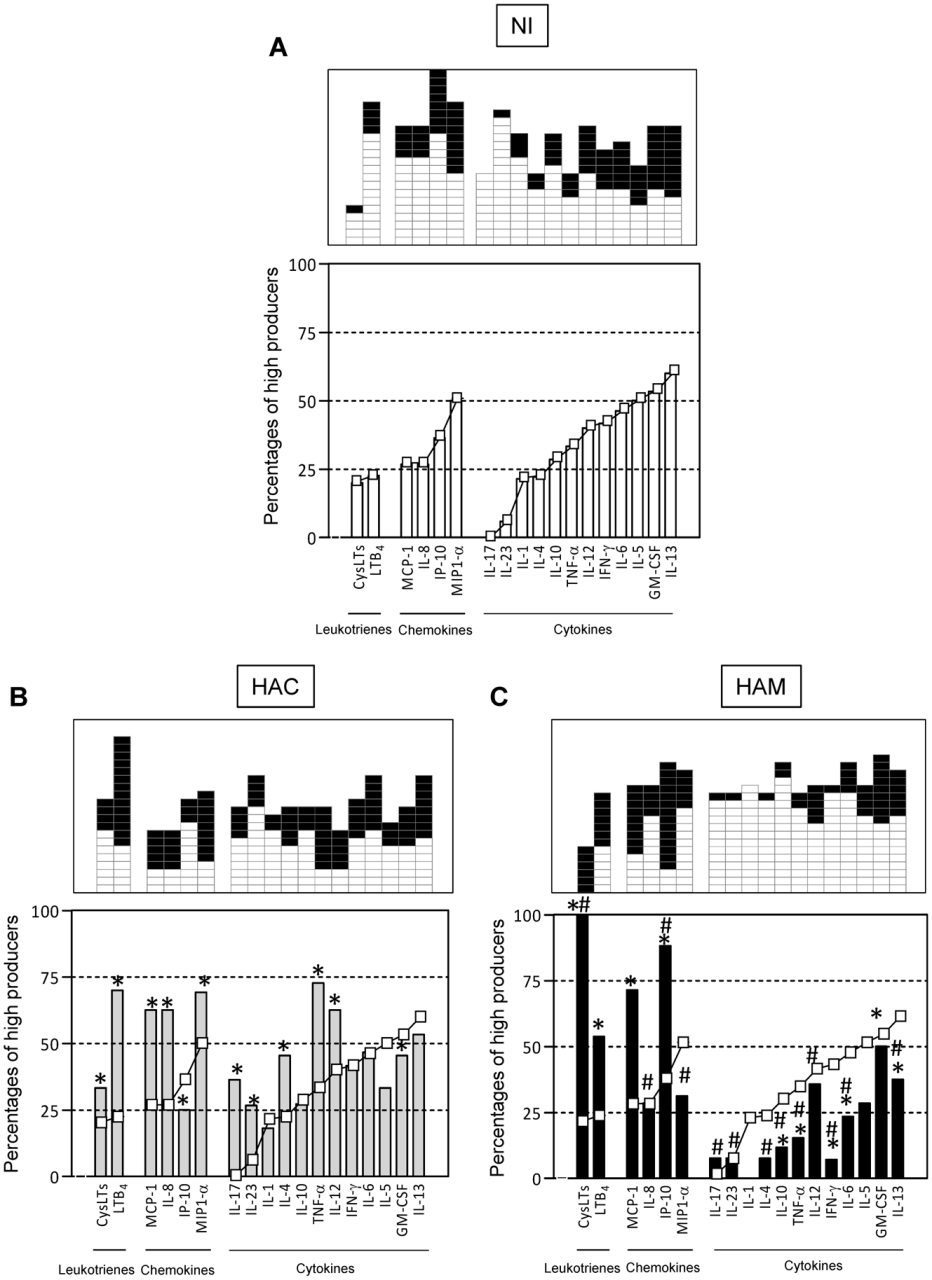
Signature curves of high biomarker producers during HTLV-1 infection. (A) Representative scattergraphs were used to establish the concept of low biomarker producers (white) and high biomarker producers (black). The results from all groups studied (non-infected donors, HACs and HAM/TSP patients) were assembled to calculate the global median for each biomarker. Low biomarker producers were defined as having values lower than the global median, whereas high biomarker producers were defined as having values greater than or equal to the global median cut-off. Data from 3 of 18 molecules analyzed are shown. (B–D) The diagrams were plotted using the global median index of plasma biomarkers (measured by ELISA) as the cut-off to identify each volunteer as a low (□) or high (▪) producer. The ascendant frequency of high producers found in the NI group was established as a reference curve (–□–) to identify changes in the overall biomarker signature of the other groups. Significant differences were defined as a shift to a distinct 25% quartile interval between the studied groups. *, significantly different values compared with NI donors; ^#^, significantly different values compared with HACs. NI – non-infected healthy donors; HAC – HTLV-1 asymptomatic carriers; HAM – HAM/TSP subjects.

We next sought to characterize the immune and inflammatory mediators in the plasma of NI donors to allow for further comparative analysis of HACs and HAM/TSP patients. We assessed the overall LT, chemokine and cytokine signatures by categorizing volunteers as “low-” or “high-” mediator producers to minimize the impact of individual concentrations on the final analysis and to make the data more homogeneous. The global median index of each mediator was calculated (CysLTs = 438.9; LTB_4_ = 402.6; IP-10 = 91.4; MCP-1 = 75.8; MIP1-α = 41.2; IL-8 = 0; IL-17 = 0; IL-23 = 0; IL-1 = 0; IL-4 = 0; IL-10 = 0; TNF-α = 0; IL-12 = 0; IFN-γ = 0; IL-6 = 0; IL-5 = 0; GM-CSF = 9.2; IL-13 = 26.2) (data not shown), and based on these values, each volunteer was classified as a low- (□) or high (▪)-mediator producer (upper panels in Figures 4 A, B, C). An assembly of the frequency of high-mediator producers among NI donors in ascendant fashion is shown in Figure 4A. The mediator signature curves of NI donors were used as a reference to identify changes in the overall mediator signatures of HACs and HAM/TSP patients. Analysis of the HAC signatures demonstrated that LTs, the majority of chemokines (MCP-1, IL-8 and MIP1-α) and some cytokines (IL-17, IL-23, IL-4, TNF-α and IL-12) are increased when compared to the values observed in NI donors (Figure 4B). We also examined the signatures of HAM/TSP patients (Figure 4C) and found that LTs and chemokines (MCP-1 and IP-10) were increased, and in contrast to our findings in HACs, cytokines were decreased when compared to the values observed in NI donors. Additionally, high producers of CysLTs and IP-10 were more frequent in HAM/TSP group than in HACs. In contrast, the frequency of high cytokine producers was lower in HAM/TSP patients than in HACs. Thus, our findings showed that LTs and chemokines are the prominent mediators in HACs and HAM/TSP patients.

### Association between Immune and Inflammatory Mediators in HTLV-1 Infection

**Figure 5:**
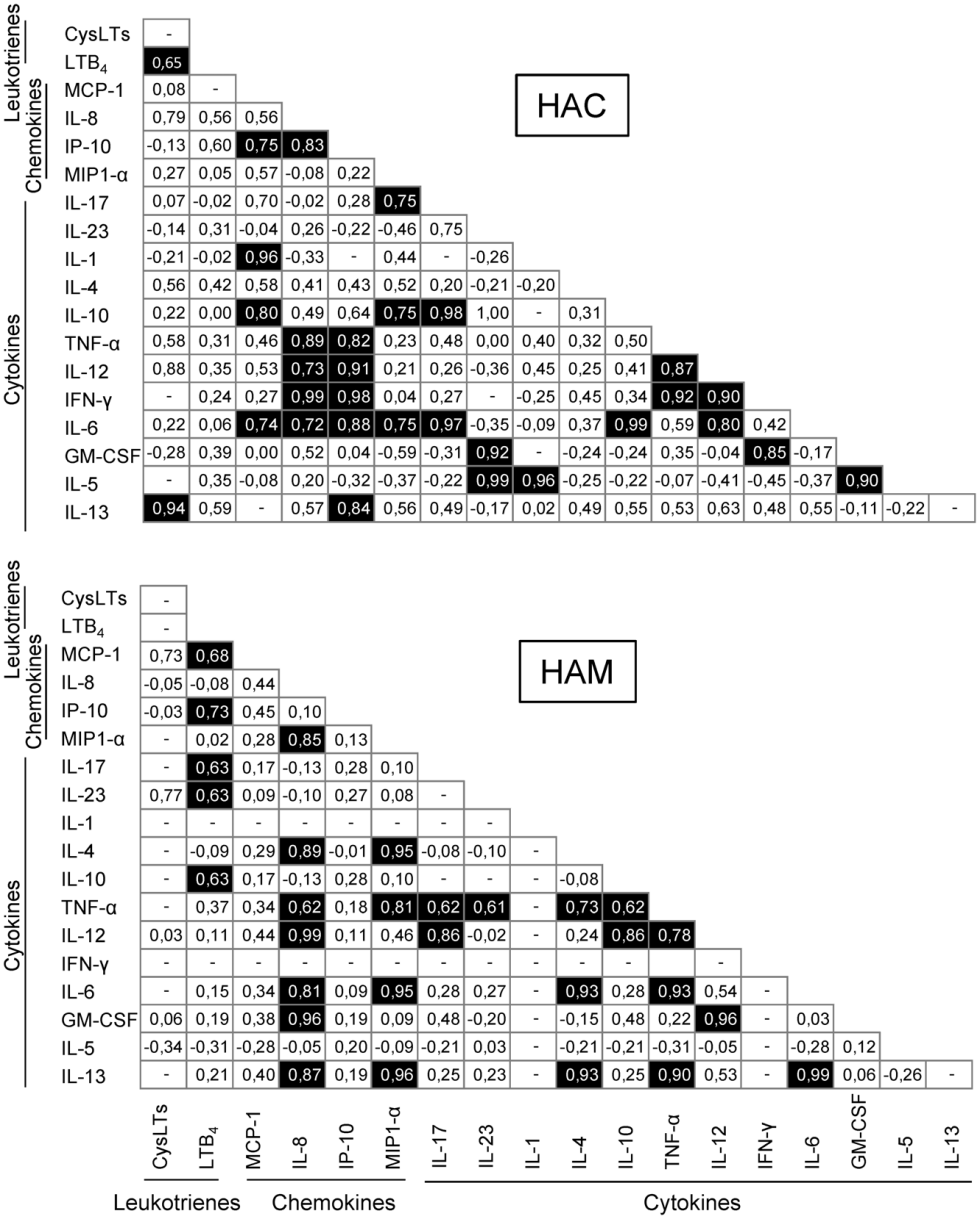
Correlation of plasma biomarkers in HTLV-1 infection. Samples from HTLV-1-infected subjects were used to detect leukotrienes, chemokines and cytokines by ELISA. A correlation analysis was performed to analyze biomarkers levels. The results of a non-parametric Spearman’s test and ‘*r’* index are provided in the figure. Filled squares indicate positive correlations. HAC – HTLV-1 asymptomatic carriers; HAM – HAM/TSP subjects.

The differences in the concentrations of LTs, chemokines and cytokines between HACs and HAM patients prompted us to investigate the correlation between the concentrations of mediators in each group. The analysis of the HAC group demonstrated positive correlation between the concentrations of CysLTs with the concentrations of LTB_4_ and IL-13 (Figure 5A). In contrast to our findings in HACs, CysLT concentrations were not correlated with the amounts of other mediators, but LTB_4_ concentrations were positively correlated with the levels of some chemokines, including MCP-1 and IP-10, and cytokines, including IL-17, IL-23 and IL-10 in HAM/TSP patients (Figure 5B). Meanwhile, although no specific pattern associated with any kind of immune or inflammatory response was observed, the expression levels of several chemokines and cytokines were correlated in HACs and HAM/TSP patients (Figure 5).

## Discussion

The participation of LTs in several infections [19], [20] and inflammatory disorders [21], [22] has long been appreciated; however, the involvement of these lipid mediators in HTLV-1 infection and HAM/TSP development has not been studied previously. Here, we report for the first time that HTLV-1 infection dysregulates the LT pathway. Our results demonstrate increased LTB_4_ and CysLT plasma concentrations in HTLV-1 patients, suggesting a role for LTs in several HTLV-1-associated inflammatory diseases. Furthermore, a key finding in our study was the association between plasma CysLT concentrations and HAM/TSP. The concentration of plasma CysLTs was increased more than 3-fold in HAM/TSP patients when compared to HACs. Studies have detected LTs in the central nervous system of patients with autoimmune diseases [23] and infectious diseases [24], [25] and have suggested a potential pathophysiological role for these molecules. Specifically, the inhibition of 5-LO activity during experimental demyelination attenuates neuroinflammation and axonal damage [26]. Together, these observations are consistent with our results demonstrating that HAM/TSP patients display enhanced CysLT production, suggesting that these mediators contribute to HAM/TSP pathogenesis. As there is no effective therapy for HAM/TSP [27], CysLT signaling may represent a new therapeutic target. Although many investigators have concentrated their efforts on the discovery of HAM/TSP markers, previous studies have relied on ex vivo culture, and few associations have been established in vivo [28], [29]. Thus, our work extends to the knowledge of in vivo HAM/TSP markers by presenting CysLTs as a putative biomarker of HAM/TSP. Therefore, we next tested the hypothesis that HTLV-1 proviral load is correlated with the concentration of plasma LTs. Using Pearson’s correlation, we observed a positive correlation between LTB_4_ or CysLTs and proviral load indicating that concentrations of LTs in plasma of infected individuals reflect proviral load. However, in the present study, our data did not demonstrate a strong association between LTs and disease activity or even clinical progression in HAM/TSP patients. In this pioneering investigation, we explored the complex pro-inflammatory network underlying the immunological profile of HLTV infected patients to find potential biomarkers of disease activity or even prognostic markers for monitoring purposes. We believe that LTs could be putative immunological biomarkers that could serve as prognostic markers or could be associated with disease activity. It is important to mention that the present investigation should be considered the first step toward the discovery of LT biomarkers for HLTV infection, as further studies will be necessary to validate this hypothesis.

LTs are produced primarily by neutrophils, eosinophils, mast cells and monocytes/macrophages [30]. Among PBMCs, it is assumed that monocytes are largely responsible for LT generation. Moreover, B cells express 5-LO but do not synthesize LTs upon A23187 stimulation [31]. Notably, we found that cell-free viral particles induce LT generation by PBMCs after A23187 stimulation, suggesting that monocytes are a significant source of LTs during HTLV-1 infection. This also suggests that LT production is increased in the central nervous system of HAM/TSP patients because infiltrating monocytes are found in the areas of demyelination [4] and may be stimulated by the virus to release lipid mediators. Importantly, HTLV-1 and HTLV-1-Tax antigen induce another lipid mediator, PGE_2_ [32], demonstrating that HTLV-1 regulates both the 5-LO and the cyclooxygenase pathway of the AA cascade. Moreover, LTC_4_ generation by PBMCs supports elevated concentrations of plasma CysLTs in HAM/TSP patients. However, despite the low number of HTLV-1 virions in vivo, a DNA insertion analysis showed that the virions can induce the production of LTs in PBMCs in vitro without infecting these cells (data not shown). On the other hand, LTs are involved in the control of host defense, including defense against HIV [33], [34]; thus, the decreased LTB_4_ generation observed in the PBMCs of HAM/TSP patients when compared to HACs may not represent a good method for controlling the high HTLV-1 set points seen in HAM/TSP patients. In addition, it was interesting to note the lack of correlation between 5-LO and LTC_4_S gene expression and LT generation by human PBMCs. Despite this dissociation between changes in 5-LO mRNA levels and protein expression, which has been reported previously [35], [36], increased 5-LO mRNA expression indicates a positive regulation of the LT pathway as evidenced by the increased mRNA expression of 5-LO in HAM/TSP PBMCs.

The upregulation of LT receptors has been noted previously in experimental neuroinflammatory disease and is thought to be involved in the pathogenesis of this disease [37], [38]. It is noteworthy that LT receptor expression has been detected in both CD4^+^ and CD8^+^ T cells [12]. These cells are found in inflamed areas in HAM/TSP patients. Thus, we hypothesized that LT receptor expression may be increased in T cells in HAM/TSP patients. Our data demonstrate that gene expression of LT receptors is modulated by HTLV-1 infection. Specifically, BLT_1_ was upregulated in CD4^+^ T cells from HAM/TSP patients. Meanwhile, CysLT_1_ was upregulated in CD4^+^ T cells but downregulated in CD8^+^ T cells of HACs and HAM/TSP patients. In animal models, deletion of BLT_1_ [39] and inhibition of CysLT_1_ [40] signaling can suppress the recruitment of inflammatory cells into the central nervous system and thus inhibit experimental autoimmune encephalomyelitis. In this regard, we speculate that high LT amounts and high LT receptor expression levels in CD4^+^ T cells may bias the host toward cellular infiltration of inflamed tissues, worsening the HAM/TSP disease. Moreover, as HTLV-1 preferentially affects CD4^+^ T cells, the migration of non-infected CD4^+^ T cells to inflamed sites containing HTLV-1-infected lymphocytes could facilitate cell-cell contact and consequently the spread of infection.

Studying the immunological response to HTLV-1 infection is important for the understanding of HAM/TSP pathogenesis. Others have shown that CXCL9 [41], CXCL10 [42], CCL22 [43], IP-10 [28], sCD30 [28] and IFN-γ [28] are increased in the systemic circulation of HTLV-1-infected individuals. We hypothesized that dysregulation of the immune system is likely to be involved in the pathogenesis of HLTV-1 infection and that the clinical presentation of HAM/TSP may result from multifactorial immunological mechanisms. Impairment in the cytokine network has been found to be one of the determining factors in several human diseases. Because conventional strategies may not be suitable to capture minor changes in the immunological profile and because of the wide range of chemokines/cytokines/LTs, in this study, we employed an alternative strategy to assess the biomarker signature and to describe the dominant profiles associated with asymptomatic presentation and HAM/TSP caused by chronic HTLV-1 infection. This panoramic overview offers additional insight into the immunological events that are relevant for clinical studies of HTLV-1 infection. This approach may allow for a better understanding of the immunological parameters that control disease outcome and provide useful tool for prognostic monitoring. We therefore examined the concentrations of several chemokines and cytokines in the plasma of our cohort to further establish a signature curve for LTs, chemokines and cytokines. Using a signature curve of non-infected individuals as a reference, we demonstrated that LTs and chemokines are increased in the plasma of both HACs and HAM/TSP patients. The signature curves both confirm CysLT and identify IP-10 as biomarkers of HAM/TSP. The Th1-associated chemokine IP-10 belongs to the CXC chemokine superfamily. IP-10 has been shown to be a potential marker of inflammation and diseases [43]–[45] including HAM/TSP [28]. Furthermore, our results show that although the plasma concentrations of some cytokines are increased in HACs, the majority of the analyzed cytokines are decreased in the plasma of HAM/TSP patients. The low levels of these cytokines observed in HAM/TSP patients may reflect the attenuated inflammatory response observed in the central nervous system after a long period of HAM/TSP manifestation [46]. In contrast to these decreased cytokine levels, however, our work clearly demonstrates some plasma mediators of inflammation remain elevated even after a long period of disease manifestation. In addition, as a trigger for positive feedback regulation, LTs, chemokines and cytokines have been shown to influence the production of each other [16], [18], [47], [48]. Supporting these findings, we have shown positive correlations between plasma LT, chemokine and cytokine concentrations but in the absence of a singular pattern of immune response. Importantly, CysLTs are increased in HAM/TSP patients, but no positive correlation was detected between CysLT levels and the concentrations of other mediators, presenting this family of lipid mediators as an independent biomarker of HAM/TSP.

In this report, we demonstrate that HTLV-1 dysregulates the LT pathway. This finding has important implications for the understanding of HAM/TSP. First, CysLTs could be used as a biomarker for HAM/TSP development. Second, the fact that LTs are mediators of inflammation suggests that the LT pathway might be involved in HAM/TSP pathogenesis. Therefore, further experiments will be required to elucidate a potentially pathogenic function of LTs in HAM/TSP. Moreover, it will be interesting to determine whether drugs targeting the LT pathway ameliorate HAM/TSP symptoms.

## Patients, Materials and Methods

### Study Population

The study subjects were classified as asymptomatic HTLV-1 carriers or HAM/TSP patients in accordance with the criteria proposed by the World Health Organization. Non-infected (NI) healthy volunteers were included as a control (Table 1). Biological specimens were obtained from HTLV-1 patients from the clinical cohort of the Neurology Department of Ribeirão Preto University Hospital, Brazil. Diagnosis of HTLV-1 infection was established by enzyme-linked immunosorbent assay (ELISA) (rp21e-enhanced EIA; Cambridge Biotech Corp.) and confirmed by PCR **(**tax and LTR regions). Subjects with HAM/TSP were selected from among a heterogeneous disease progression group. Individuals receiving therapies were excluded. All procedures were approved by the Ethical Committee of the University Hospital, School of Medicine of Ribeirão Preto, University of São Paulo (process number 1108/2008), and all subjects provided written informed consent.

**Table 1:** Demographical and Clinical Characteristics of the Study Groups.

Group	Number	Age, mean years (± SD)	Sex (M/F)^a^	Proviral load mean/10^5^ cell (± SD)	First reported symptoms, mean years (± SD)
Non-infected	31	45.7 (±12.2)	9/22	N/A^b^	N/A
HAC^c^	26	42.9 (±12.9)	11/15	2,105.4 (±2,312.2)	N/A
HAM/TSP^d^	19	54.9 (±9.1)	4/15	6,180.9 (±3,544.6)	13 (±9.7)

**Note.** ^a^M/F: male/female;

b N/A: not applicable;

c HAC: HTLV-1 asymptomatic carriers;

d HAM/TSP: HTLV-1 associated myelopathy/tropical spastic paraparesis.

### Isolation of Blood Leukocytes

After separation of plasma from the heparinized venous blood, PBMCs were isolated using Ficoll-Paque (GE Healthcare) density gradient centrifugation. To isolate lymphocytes from PBMCs, magnetic beads conjugated with anti-CD4 or anti-CD8 antibodies (Mini-Macs Micro-Beads, Miltenyi Biotec) were used to separate CD4^+^ and CD8^+^ T cells by positive selection using the manufacturer’s protocol. Phenotypic analysis performed by flow cytometry (BD-FACSCanto) using anti-CD4-FITC, anti-CD8-FITC and anti-CD3-PE antibodies (BD Biosciences) demonstrated a minimum of 80% purity of CD4^+^ and CD8^+^ lymphocytes. A hemocytometer chamber was used to obtain absolute cell counts, and cell viability was determined by trypan blue exclusion.

### HTLV-1 Proviral load

HTLV-1 proviral load was quantified as previously described [49]. Genomic DNA samples isolated from peripheral blood of the HTLV-1 infected individuals were used to perform quantitative RT-PCR with the SYBR Green system (Applied Biosystems). The single-sample reactions for human *β*-actin and HTLV-1 tax were performed in duplicate on the same plate. The HTLV-1 proviral load was calculated using the following equation: average of tax/average of *β*-actin×2×10^5^. The values obtained were Log10 transformed for the correlation analysis.

### Detection of Leukotriene Pathway Transcripts

Total RNA was extracted from PBMCs and lymphocytes with TRIzol (Invitrogen) according to the manufacturer’s instructions, and reverse transcription was carried out with 2 µg of total cellular RNA using a High-Capacity cDNA Reverse Transcription kit (Applied Biosystems). Thereafter, quantitative RT-PCR was performed using ABI 7500 Sequence Detection (Applied Biosystems). The reaction was performed in duplicate using TaqMan assay reagents (Applied Biosystems) (product reference for 5-LO: Hs_00386528_m1; LTC_4_ synthase: Hs_00168529_m1; BLT1: Hs_00175124_m1; CysLT1: Hs_00929113_m1) and analyzed using 7500 System SDS software. The relative mRNA expression was determined using the ΔΔCT method. Glyceraldehyde 3-phosphate dehydrogenase (GAPDH: 4310884-E) was used as an internal control for PBMCs. The geometric means of the values obtained for β-actin (ACTB: 4326315E), GAPDH, β2 microglobulin (B2M: 4333766-0710013) and ribosomal protein L13a (RPL13a: 185720330-7) were used as internal controls for CD4^+^ T cells, and ACTB was used as an internal control for CD8^+^ T cells.

### Production of Cell-free Virus

The human T cell line MT-2 was used as a source of HTLV-1 producing cells. For preparation of cell-free HTLV-I, cells were seeded at 5×10^5^/mL and incubated at 37°C in a humidified CO_2_ atmosphere for 2 days in RPMI supplemented with 10% FBS. The supernatants were passed through a 0.45-µm filter (Millipore) to remove cells and debris, and the virions were concentrated 10 times by ultracentrifugation for 2 hours at 100,000 g. The pellet containing virus particles was resuspended in RPMI and quantified before being subjected to HTLV-1 p19 ELISA (ZeptoMetrix).

### Stimulation and Culture of Cells

We plated PBMCs (10^6^/well) in forty eight-well plates and maintained them overnight at 37°C and 5% CO_2_. The cells were provided with fresh RPMI 1640 containing 5% AB human serum (Sigma) and 100 U/mL penicillin and cultured for an additional 48 hours. For analysis of the effects of cell-free HTLV-1, cell cultures from healthy donors were challenged with 10 ng of virions particles (p19 equivalent) prior additional culture. For leukotriene detection, the supernatants were removed, and the cells were resuspended in HBSS containing Ca^2+^ and Mg^2+^ and stimulated for 30 minutes with 0.5 µM of the calcium ionophore A23187 (Sigma), and then, reactions were stopped using ice.

### Measurement of Leukotrienes, Chemokines and Cytokines

A specific enzyme immunoassay (Cayman) was used to quantify LTB_4_ and LTC_4_ in cell-free supernatants and LTB_4_ and CysLT in plasma per the manufacturer’s instructions. For plasma measurements, samples stored at −70°C were purified on Waters C18 Sep-Pak cartridges (Waters Associates) prior to performing the assay. Moreover, the cell-free supernatants were tested for IP-10 and TNF-α, and the plasma samples were tested for MCP-1, MIP1-α, IP-10, IL-8, IL-5, IL-4, IL-13, IL-1, IL-6, GM-CSF, TNF-α, IL-12, IFN-γ, and IL-10 using a Duoset ELISA Development kit (R&D Systems) and for IL-17 and IL-23 using an OptEIA ELISA kit (BD Bioscience) in accordance with the manufacturer’s instructions. The reactions were performed in 96-well ELISA plates (Corning), and the optical densities were determined at 450 nm using a microplate reader. The cytokine concentration in each sample was estimated by interpolation of sample optical densities with the cytokine standard using a four-parameter curve-fitting program.

### Leukotriene, Chemokine and Cytokine Signature Analysis

A method for identifying low and high producers of mediators by analyzing cytokine profiles was previously reported by Luiza-Silva et al. [50]. The concentrations of LTs, chemokines and cytokines (pg/mL) were assembled to calculate the global median index ([values of NI donors+HACs+HAM/TSP patients]/number of samples), and plasma samples were characterized as low- or high-mediator producers. Low-mediator producers were defined as having values lower than the global median, whereas high-mediator producers were defined as having values greater than or equal to the global median cut-off. The percentage of high producers was calculated for each analyzed molecule, and the ascendant frequency of the non-infected group was used as the reference curve to identify changes in the overall mediator patterns from all the groups.

### Statistics

The data are presented as means ± SEM of values determined from the indicated number of samples. The data were analyzed by Student’s *t-*tests or ANOVA with Bonferroni’s post-test as appropriate to identify significant differences between group means using GraphPad Prism version 5 (GraphPad Software).

Spearman’s correlation test was performed to assess the association between the levels (pg/mL) of LTs, chemokines and cytokines while Person’s test was used to analyze the association of LTs and the HTLV-1 proviral load. In all cases, statistical significance was defined as p≤0.05. The cytokine signatures analyses were performed using the non-infected signature as the reference curve, and differences were considered significant when the values fell outside of the quartile of the reference signature. The use of the 50th percentile as the limit to identify relevant differences in the chemokine/cytokine/LT signatures between the groups has been adapted from a pioneering study by Luiza-Silva et al. [50]. This approach has been shown to be relevant to detect, with high sensitivity, putative minor changes in the cytokine signatures that are not detectable by conventional statistical approaches.
